# Mitochondrial Cytochrome c Oxidase Subunit 1 Sequence Variation in Prostate Cancer

**DOI:** 10.6064/2012/701810

**Published:** 2012-05-14

**Authors:** Takara A. Scott, Rebecca S. Arnold, John A. Petros

**Affiliations:** ^1^Department of Urology, Emory University, Atlanta, GA 30322, USA; ^2^Atlanta VA Medical Center, Decatur, GA 30033, USA; ^3^Department of Pathology and Laboratory Medicine, Emory University, Atlanta, GA 30322, USA; ^4^Department of Hematology & Medical Oncology, Emory University, Atlanta, GA 30322, USA

## Abstract

*Purpose*. Mitochondrial DNA (mtDNA) mutations have been described in every adult neoplasm including prostate cancer. There are marked racial differences in mutations within the cytochrome c oxidase subunit 1 (COI) gene in individuals with prostate cancer (PCa). The purpose of this study was to identify the variation in COI gene sequence in African and Caucasian Americans with prostate cancer. *Methods*. We sequenced the COI gene from peripheral blood in 482 prostate cancer patients and 189 controls. All bases that differed from the revised Cambridge Reference Sequence (rCRS) were classified as either silent or missense and the compiled alterations were then compared between races and published reports. *Results and Conclusions*. We found inherited mtDNA COI missense variants in 8.8% of Caucasian prostate cancer patients (vs. 0.0% controls) and 72.8% of African-American prostate cancer patients (vs. 64.3% controls) A total of 144 COI variants were identified, of which 30 were missense mutations. Of 482 PCa patients, 116 (24.1%) had one or more missense mutations. Further evaluation of this gene and these mutations may allow for the identification of genetically at-risk populations. The high rate of COI mutations in African-Americans may account for some of the racial disparity observed in prostate cancer.

## 1. Introduction

Prostate cancer is the second most common cause of cancer death among men in the United States [1] with African American men 2.4 times more likely than Caucasian men to die from this disease, likely due to both genetic and environmental factors [2]. 

Mitochondria are found in all cells and are central to energy production, reactive oxygen species generation, and apoptosis, all altered in cancer. The mitochondrion is the site of cellular ATP production during the process of oxidative phosphorylation that involves the electron-transport chain (respiratory complexes I–IV) and the ATP synthase (complex V). The mitochondrion contains its own DNA (mtDNA), a 16.5 kb circular self-sufficient intron-free molecule that encodes two ribosomal RNAs (12S and 16S rRNAs), a complete complement of 22 transfer RNAs (tRNAs), and 13 polypeptides. mtDNA mutations have been found in breast, colorectal, ovarian, gastric, lung, pancreatic, brain, renal, thyroid, and many other solid tumors, including prostate [3]. MtDNA mutations have also been found in Lebers' hereditary optic neuropathy, Leigh syndrome, diabetes, Alzheimer disease, and Parkinson's disease [4]. 

MtDNA with its high copy number, is maternally transmitted, lacks recombination, and has a higher sequence evolution rate than the nuclear genome [5]. Consequently, human populations, from discrete maternal lineages, harbor unique sets of mtDNA single nucleotide polymorphisms (SNPs) that define particular genetic backgrounds referred to as haplogroups [6, 7]. SNPs in protein coding regions occur frequently in the human genome and can be classified as: missense (amino acid altering) or silent [8].

 A major challenge lies in determining whether observed mutations are pathogenic. Amino acid altering mutations may affect protein function or may be essentially neutral [9].

 Previously, we found that germline mutations in the mtDNA gene cytochrome c oxidase subunit 1 (COI) were associated with prostate cancer in Caucasian men [10]. We also reported that two mutations T6221C and T7389C were associated with PCa in African American men [11]. There are marked racial differences in specific inherited COI gene mutations linked to prostate cancer. We therefore sequenced COI genes in prostate cancer cases and controls and compared mutations between ethnic groups.

## 2. Materials and Methods

### 2.1. Subjects

Some of the study subjects were described previously [10, 11]. In addition, new patients were enrolled prior to radical prostatectomy and included in this paper. All experiments are covered by an Emory IRB approved protocol.

The “no-cancer” control group was subjects at least 50 years old found to be free of prostate cancer as previously described [10, 11]. Caucasian controls and a small proportion of African American controls had undergone prostate biopsy to document the absence of prostate cancer. African American controls were followed for approximately 5 years and found not to have developed prostate cancer.

### 2.2. Preparation of Genomic DNA/Polymerase Chain Reaction (PCR) Amplification

Samples from prostate cancer patients were selected from Emory's tissue bank, and peripheral blood mononuclear cell DNA was extracted using Qiagen FlexiGene (Valencia, CA, USA). For amplification of the mitochondrial COI region 5904–7445, the following sets of primers were used: **5772F**: 5′AGGTTTGAAGCTTCTTC3′; **6720R**: 5′TACCTATGTATCCAAATGGT3′ and **6531F**: 5′CTAACAGACCGCAACCTCAA3′; **7620R**: 5′GCGTCTTGTAGACCTACTTG3′ (IDT, Coralville, IA, USA). Each PCR reaction was performed in 50 uL containing between 50–150 ng DNA, 0.2 mM each dNTP, 1.5 mM MgCl_2_, 0.15 mM each primer (Roche, Indianapolis, IN, USA), and 2.5 units of AmpliTaq Gold DNA Polymerase (Applied Biosystems, Foster City, CA, USA). The reaction conditions were: 95°C for 7 min, followed by 40 cycles of amplification at 94°C for 1 min, 55°C for 1 min, and 72°C for 1 min. Double-stranded PCR products were visualized by ethidium bromide staining of agarose gels.

### 2.3. mtDNA COI Gene Sequencing

Sequencing was performed using BigDye Terminator Cycle Sequencing Kit (Applied Biosystems, Foster City, CA, USA) in 20 *μ*L containing PCR product pretreated with ExoSAP-IT (USB, Cleveland, OH) and eight sequencing primers from IDT: (5772F, 6720R, 6531F, 7620R, (**6080F**: 5′TCTACAACGTTATCGTCACA3′), (**6930F**: 5′TGCAGTGCTCTGAGCCCTAG3′), (**6340R**: 5′CTAGGTGTAAGGAGATG3′), and (**7150R**: 5′GATTTACGCCGATGAATATG3′)). The templates were denatured at 96°C for 1 min, followed by 25 cycles of 96°C for 10 sec, 55°C for 5 sec, and 60°C for 4 min. Excess dye terminators were removed using Centri-Sep 96 plates (Princeton Separation, Adelphia, NJ, USA) and resuspended with 20 *μ*L of Hi-Di Formamide (Applied Biosystems, Warrington, UK). Sequencing was performed using an Applied Biosystems PRISM 3100 Genetic Analyzer and analyzed by SEQSCAPE V2.1. All nucleotide substitutions were compared to the Cambridge Reference Sequence (rCRS).

### 2.4. Characterizing Pathogenicity of Mutations

#### 2.4.1. Conservation Index (CI), Grantham Value (GV), and Allelic Index (AI)

Evolutionary conservation is a strong predictor of pathogenicity with amino acid substitutions at evolutionarily conserved positions more likely to be pathogenic than those at less conserved positions. We compared the amino acid sequence of the COI gene of 61 nonhuman mammalian species to determine the conservation index of the 30 missense mutations identified in this study [12]. The conservation index (CI) was calculated by determining the percentage of these 61 species for which the amino acid is the same as the wildtype human amino acid. The Grantham value (GV) was calculated in order to evaluate the difference in composition, polarity, and molecular volume of mutant and wildtype amino acids [13]. The frequency of each mutation was compared to the mutation's frequency in various human populations such as the online mtDB database of 2,704 individuals [14]. We have defined the “allelic index” of any base substitution as the percentage of the 2,704 sequences in the mtDB with that mutation. If there are no reports in the database, then the allelic index is reported either as “unique” or having an allelic index of zero.

### 2.5. Classification of Pathogenicity Using Computer Algorithms

We used three programs to classify the pathogenicity of 30 missense mutations. (i) Polymorphism Phenotyping v2 (PolyPhen2) is software that uses eight sequence-based alignments and three structure-based criteria to predict the impact of amino acid substitutions on the structure and function of proteins using physical and evolutionary comparisons. A mutation is appraised as benign, possibly damaging, or probably damaging [15, 16]. (ii) Nonsynonymous single-nucleotide polymorphism Analyzer (nsSNP) is another tool that uses information contained in multiple sequence alignment and 3D protein structure to make predictions. The mutation is appraised as neutral, disease, or unknown (when lack of data prohibits prediction). nsSNP analyzer, incorporating the Sorting Intolerant From Tolerant (SIFT) server, calculates three types of information: (1) the structural environment of the SNP, including the solvent accessibility, environmental polarity, and secondary structure; (2) the normalized probability of the substitution in the multiple sequence alignment; (3) the similarity and dissimilarity between the original amino acid and mutated amino acid [17]. (iii) PMUT is a server devoted to the prediction of the pathological character of single amino acid substitution that works at two different levels: (1) it retrieves information from a database of mutational hotspots and (2) it analyzes SNPs. The method provides a reliability index ranging between 0 (low) and 9 (very reliable) [18].

## 3. Results

We analyzed complete mtDNA COI gene sequences in 482 prostate cancer patients, including 250 Caucasians and 232 African Americans. We have also sequenced 46 Caucasian and 143 African American “no-cancer” controls. We found a total of 192 inherited mtDNA COI missense variants in 8.8% of Caucasian prostate cancer patients (versus 0.0% controls) and 72.8% of African American prostate cancer patients (versus 64.3% in controls; Table 1). 

A total of 144 COI sequence differences with respect to the rCRS were identified including 30 missense variants (Table 3). Fisher's exact test was used to evaluate the association between the sum of COI missense mutations per prostate cancer patient versus the “no-cancer” control. A total of 116 patients exhibited at least one missense mutation in this gene at 30 distinct loci. Overall, when race was not considered, the mutation rate between cases and controls (*P* = 0.424) was similar; however, race-specific mutation rates did reveal a statistically significant difference in cases and controls in Caucasians (Table 1).

Twelve of the 30 missense mutations are highly conserved with a CI of at least 97%–100%: A5935G, G5949A, G5973A, G6081A, T6124C, G6261A, G6285A, A6663G, G6924T, G7041A, T7080C, and A7305C (Table 2). Conversely, the following mutations: C5911T, G5913A, A6040G, T6253C, C6340T, A6891G, A7083G, A7146G, C7147T, A7158G, T7354C, and T7389C changed nonconserved amino acids. A heteroplasmic mutation was found at position A6485A/G in one patient that is a synonymous mutation. 

## 4. Discussion

We presented the first evidence in 2005 that inherited mutations in the mitochondrial COI gene predispose to prostate cancer in a predominantly Caucasian American population [10]. In 2009 we studied African Americans and also found inherited mutations in this gene [11]. In comparing these two groups, we found both interesting similarities and striking differences. The object of this paper is to provide a combined analysis of these findings along with an additional 89 new prostate cancer patients in order to better understand the variation in this gene and correlation with ethnicity in prostate cancer. Thus we now report the combined analysis that includes 482 cases and 189 controls for a total of 671 individuals that have had complete COI gene sequencing.

### 4.1. COI Missense Mutations Pathogenicity in Prostate Cancer

Amongst amino acid altering (missense) mutations (relative to Cambridge), one can begin to assess the likelihood that the substitution is biologically important by two main methods. The first is analysis of the degree to which the amino acid side chains differ in terms of chemical composition, polarity, and molecular volume. For the Grantham value (GV), lower numbers indicate a relatively mild chemical difference (thus less likely to be important biologically) and higher numbers indicate a relatively marked chemical difference (more likely to be biologically important; Table 2). The second (and more common) method of assessing the likely biologic importance of the amino acid substitution is to calculate the interspecies conservation index (CI). If all 61 nonhuman mammals have the wildtype amino acid, then the CI is 100 and the amino acid is highly conserved from an evolutionary perspective and highly likely to be pathogenic.

The next level of analysis is to compare the rate of that specific mutation in various populations including prostate cancer cases and controls or a comparison of prostate cancer cases to larger population databases of sequences. The online mtDB database contains 2,704 individual complete mitochondrial DNA sequences and is readily searched [14]. Because there is no clinical or disease information for these 2,704 individuals, some are men and some are women, and some of the men undoubtedly either had prostate cancer or would develop prostate cancer, this comparison differs fundamentally from a comparison of mutation frequency in cases and controls. The advantage to this population databases is that there are a large number of sequences (individuals) with which to compare mutation frequencies. The disadvantage is that there is no associated clinical information, specifically prostate cancer incidence.

We also compared the frequency of each mutation in cases and controls. Our data includes two distinct case-control comparisons. The first is the Caucasian American (CA) cases (*N* = 250) that are compared to a CA control group that was rigorously defined as *not* having prostate cancer (*N* = 46). These controls were recruited from a prostate biopsy cohort and fulfilled the following criteria: they were men of at least 50 years of age that had a serum PSA less than 4 ng/mL and at least one set of negative prostate biopsies. The second case-control comparison is drawn from the Flint Men's Health Study and is restricted to AAs. The cases were all pathologically verified, and the controls were AA men between the ages of 40 and 79 living in Genesee County, MI, USA that had serum PSA below 4.0 and a negative digital rectal exam (not biopsy). Added to this control group were 9 AA men from the biopsy-negative control group. The final numbers of AA cases and controls are therefore 232 and 143, respectively. 

Our findings were both race specific and race independent. If one compares the overall frequency of missense COI mutations in all prostate cancer cases (192/482 = 39.8%) to controls (92/189 = 48.7%), there is no statistically significant difference (*P* = 0.1). In CA cases, the mutation rate is 8.8% compared to 0% in CA controls (*P* = 0.03). In AA cases, the mutation rate is 72.8% compared to 64.3% in controls (*P* = 0.26). It is interesting to compare these rates of mutation to the large (clinically uncharacterized) databases. While the online mtDB database does not classify individuals as CA or AA, it does provide sequence data based on continental origin (Africa and Europe). The COI mutation rate for Europeans in this database is 79 out of 1196 for a rate of 6.6%. The mtDB database African COI mutation rate is 142 of 249 for a rate of 57% that is comparable to our (Genesee County Michigan) AA controls (at 64%). 

Sequencing of CA prostate cancer cases revealed several COI mutations that were only found in CA cases. These included missense mutations at nucleotide positions (n.p.) 6253 (3 times), 6261 (4 times), 6663 (twice), and 7080 (twice). The frequencies of these mutations in AA men were quite different, and each of these mutations was found in the controls. When both CA and AA men were considered together, the n.p. 6253 mutation was found in 7 cases and 7 controls, the n.p. 6261 mutation was found in 8 cases and 1 one control, the n.p. 6663 in 16 cases and 7 controls, and the n.p. 7080 in just 2 cases and no controls. In addition, mutations associated with African lineage were discordant in cases and controls with the 7146 mutation found in 69 cases and 36 controls and the 7389 mutation in 57 cases and 25 controls. Thus, some mutations that appeared to be only found in Caucasian prostate cancer cases were found in the African American control population. 

The CA controls had multiple negative prostate core biopsies while the AA controls were not biopsied but had only a negative digital rectal exam of the prostate. Examination alone is significantly less specific than biopsy, so there is a greater chance that undiagnosed cases entered the AA control population. 

 There are many mutations that were more common in cases than controls even in the combined group. These include inherited missense mutations at nucleotide positions 5913, 5953, 5949, 5973, 6040, 6081, 6124, 6267, 6285, 6340, 6891, 6924, 7041, 7080, 7083, 7158, and 7305 that were *never observed in any control* and 6261 that was seen in 8 cases compared to a single African American control, for a greater than threefold increased frequency in cases over controls for the combined group.

### 4.2. G6261A Mutation (Figure 1)

 The G6261A mutation is unique with a conversation index of 100% and an allelic index of 0.5. This means that all 61 non-human mammalian species have the wildtype amino acid at this location and only 0.5% of the 2704 sequences in mtDB had this alteration. This was seen in a higher rate in both CA and AA cases than their respective control groups. This mutation therefore fulfills all of our criteria for being potentially important in the pathogenesis of prostate cancer. The G6261A has also been reported in bladder cancer patients [19]. 

In another study of specific human mtDNA clades and adaptation to different climates, the G6261A mutation was reported in one Asian and one European [20]. Still other studies have reported the G6261A mutation in the Leber hereditary optic neuropathy, diabetes mellitus type 2, hypertension, colorectal cancer, and Down's syndrome [21–26]. G6261A was also found to be associated with haplogroups T2, L3Eb, R1, J, and H [21, 27–29].

#### 4.2.1. Computer Algorithms Analysis

 We used Sift, PolyPhen2, nsSNP Analyzer, and PMut programs to analyze our 30 missense mutations and found that mutations C6340T and T7080C were both predicted as disease (nsSNP analyzer) and pathological (PMut) with a PMut reliability index of 9 and 6, respectively. These numbers are deemed as having a high level of confidence. G5973A, A6040G, G6081A, T6253C, G6261A, G6267A, G6924T, C7147T, A7299G, T7354C, and A7305C were all predicted as pathological by the PMut program with level of confidence ranging from 0 (low)–9 (high). (See Table 3.)

Overall, PolyPhen2 predicted only two mutations as “Probably Damaging”, (false positive rate of 20%). The nsSNP Analyzer predicted only five of the missense mutations as “Disease,” (false positive rate of 38%, false negative rate of 28%) [18]. The PMut program predicted fifteen of the missense mutations as “Pathological,” and it has a prediction success rate of 80% in humans [18]. Ultimately, the importance of these inherited COI missense mutations in the pathogenesis of prostate cancer cannot be fully determined by sequencing alone. The true biologic impact of these mutations must be determined in controlled functional experiments the laboratory, which is beyond the scope of this paper. 

## 5. Conclusions

Inherited missense mutations in the mitochondrially encoded COI gene are present in both Caucasian and African American prostate cancer patients, and to a lesser extent in controls. There are two missense mutations in this gene that occur with exceptionally high frequency in African Americans because they arose early in human evolution and have become “fixed” in a high proportion of the population. While pathogenicity cannot be assigned to those founder mutations, the 7389 mutation was significantly associated with disease. In a combined analysis of both Caucasians and African Americans, 20 of the 30 missense mutations occur only in men with cancer and are never found in 189 controls. Of these 20 mutations, 3 (A5935G, G5949A, and G6924T) have not been observed in 2,704 complete sequences in the mtDB database and have an interspecies (*N* = 61) conservation index of 100%. Amongst these 20 mutations, one (T6124C) is predicted by all three computer algorithms (PolyPhen, nsSNP, and PMut) to be pathological with PMut assigning the highest possible reliability score (9).

The G6261 (A120T) mutation was found in 8 cancer patients compared to a single control for a 3-fold increase in frequency in cases (1.7% versus 0.5%). Furthermore, this mutation is associated with prostate cancer in both ethnic groups, is found in only 0.5% of the online 2704 sequences, and also has 100% interspecies conservation.

Inherited COI gene missense mutations are significantly associated with prostate cancer in both Caucasians and African Americans. Some significant mutations appear to be race specific while others are race independent. Specific disease-associated mutations may warrant further study in the laboratory to determine possible mechanisms of disease association. It is possible that some of the racial disparity in prostate cancer may be due to inherited mitochondrial DNA mutations.

## Figures and Tables

**Figure 1:**
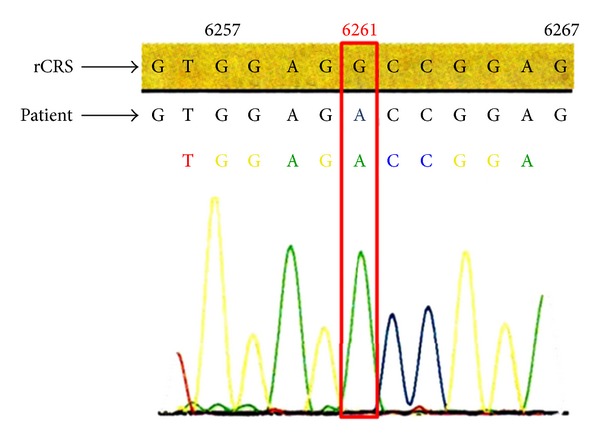
The mtDNA sequencing chromatogram shows the presence of the G→A mutation at position 6261 in one patient compared to the revised Cambridge Reference Sequence (rCRs).

**Table 1:** Frequency of COI mutations in prostate cancer cases and controls.

	*n*	COI mutant	Frequency (%)	*P* ^†^
Cancer	**482**	192	39.8	
CA	250	22	8.8	
AA	232	169	72.8	
No cancer	**189**	92	48.7	0.109
CA	46	0	0.0	0.028
AA	143	92	64.3	0.256

^†^Fisher's exact test values is represented in the right column, and frequencies were compared.

CA: Caucasian American Ancestry; AA: African American Ancestry.

**Table 2:** Missense mutations in Caucasian and African American cases and controls.

Nucleotide position	Amino acid	Conservation index	Grantham value	Allelic index	Controls (*n* = 189)	Control frequency	Cases (*n* = 482)	Case frequency
					Count	%	Count	%
C5911T	A3V	5/61 = 8%	64	0.2	4	2.1	3	0.6
G5913A	D4N	8/61 = 13%	23	0.4	0	0.0	1	0.2
A5935G	N11S	61/61 = 100%	46	Unique	0	0.0	1	0.2
G5949A	G16X	61/61 = 100%	—	Unique	0	0.0	1	0.2
G5973A	A24T	60/61 = 98%	58	0.04	0	0.0	1	0.2
A6040G	N46S	8/61 = 13%	46	0.1	0	0.0	1	0.2
G6081A	A60T	60/61 = 98%	58	Unique	0	0.0	1	0.2
T6124C	M74T	60/61 = 98%	81	Unique	0	0.0	1	0.2
G6150A	V83I	58/61 = 95%	29	0.2	8	4.2	5	1.0
T6253C	M117T	44/61 = 72%	81	0.9	7	3.7	7	1.5
G6261A	A120T	61/61 = 100%	58	0.5	1	0.5	8	1.7
G6267A	A122T	56/61 = 92%	58	0.1	0	0.0	2	0.4
G6285A	V128I	61/61 = 100%	29	0.04	0	0.0	1	0.2
C6340T	T146I	45/61 = 74%	89	0.1	0	0.0	2	0.4
G6366A	V155I	42/61 = 69%	29	0.3	1	0.5	1	0.2
G6480A	V193I	58/61 = 95%	29	0.1	0	0.0	3	0.6
A6663G	I254V	59/61 = 97%	29	0.2	7	3.7	14	2.9
A6891G	S330G	7/61 = 11%	56	0.04	0	0.0	1	0.2
G6924T	A341S	61/61 = 100%	99	Unique	0	0.0	1	0.2
G7041A	V380I	61/61 = 100%	29	0.04	0	0.0	1	0.2
T7080C	F393L	60/61 = 98%	22	0.04	0	0.0	2	0.4
A7083G	I395V	13/61 = 21%	29	0.04	0	0.0	1	0.2
A7146G	T415A	15/61 = 25%	58	3.1	36	19.0	69	14.3
C7147T	T415I*⋯*V^∗^	15/61 = 25%	89*⋯*69^∗^	Unique	0	0.0	2	0.4
A7158G	I419V	9/61 = 15%	29	0.1	0	0.0	3	0.6
A7299G	M466V	39/61 = 64%	21	0.07	1	0.5	0	0.0
T7354C	M484T	11/61 = 18%	81	Unique	1	0.5	0	0.0
A7305C	M468L	60/61 = 98%	95	Unique	0	0.0	1	0.2
T7389C	Y496H	15/61 = 25%	83	2	25	13.2	57	11.8
**G7444A**	**X514K**	—	—	0.4	1	0.5	1	0.2

					**92**		**192**

*Denotes double AA change because the patient also had a mutation at the previous AA position A7146G.

**Table 3:** Missense mutations' computation methods and prediction of phenotypic effect.

Nucleotide position	Amino acid	^ ∗^PolyPhen	^ †^nsSNP analyzer	^ ‡^PMut	^ ‡^PMut reliability
C5911T	A3V	Benign	Neutral	Neutral	3
G5913A	D4N	Benign	Neutral	Neutral	4
A5935G	N11S	Probably damaging	Disease	Pathological	**1**
G5949A	G16X	—	—	—	—
G5973A	A24T	Benign	Neutral	Pathological	**6**
A6040G	N46S	Benign	Neutral	Pathological	**1**
G6081A	A60T	Benign	Neutral	Pathological	**7 **
T6124C	M74T	Probably damaging	Disease	Pathological	**9**
G6150A	V83I	Benign	Neutral	Neutral	5
T6253C	M117T	Benign	Neutral	Pathological	**7**
G6261A	A120T	Benign	Neutral	Pathological	**7**
G6267A	A122T	Benign	Neutral	Pathological	**5**
G6285A	V128I	Benign	Disease	Neutral	2
C6340T	T146I	Benign	Disease	Pathological	**9**
G6366A	V155I	Benign	Neutral	Neutral	4
G6480A	V193I	Benign	Neutral	Neutral	3
A6663G	I254V	Benign	Neutral	Neutral	7
A6891G	S330G	Benign	Neutral	Neutral	6
G6924T	A341S	Benign	Neutral	Pathological	**1**
G7041A	V380I	Benign	Disease	Neutral	4
T7080C	F393L	Benign	Disease	Pathological	**6**
A7083G	I395V	—	—	—	—
A7146G	T415A	Benign	Neutral	Neutral	3
C7147T	T415I*⋯*V^∗^	Benign	Neutral	Pathological	**6**
A7158G	I419V	Benign	Neutral	Neutral	8
A7299G	M466V	Benign	Neutral	Pathological	**4**
T7354C	M484T	Benign	Neutral	Pathological	**0**
A7305C	M468L	Benign	Neutral	Pathological	**0**
T7389C	Y496H	Benign	Neutral	Neutral	5
**G7444A**	**X514K**	—	—	—	—

^‡^PMut: the method indicates neutral or pathological and provides a reliability index ranging between 0 (low) and 9 (very reliable).

*PolyPhen2: this method indicates probably damaging (protein function deemed affected with high confidence), possibly damaging (protein function supposedly affected), benign (most likely lacking any phenotypic effect), and unknown (lack of data do not allow PolyPhen to make prediction).

^†^nsSNP analyzer: prediction indicates neutral or disease.

## Abbreviations

mtDNA: Mitochondrial DNA
ROS: Reactive oxygen species
OXPHOS: Oxidative phosphorylation
SNPs: Single nucleotide polymorphisms
CA: Caucasian American
AA: African American
CI: Conservation index
GV: Grantham value
AI: Allelic index
rCRS: Revised Cambridge Reference Sequence.
